# Sleep problems and functional disability in children with functional gastrointestinal disorders: An examination of the potential mediating effects of physical and emotional symptoms

**DOI:** 10.1186/1471-230X-12-142

**Published:** 2012-10-15

**Authors:** Jennifer Verrill Schurman, Craig A Friesen, Hongying Dai, Caroline Elder Danda, Paul E Hyman, Jose T Cocjin

**Affiliations:** 1 The Children’s Mercy Hospital, Kansas City, MO, USA; 2 The University of Kansas Medical Center, Kansas City, KS, USA; 3 Division of Developmental & Behavioral Sciences, The Children’s Mercy Hospital, 2401 Gillham Road, Kansas City, MO, 64108, USA

**Keywords:** Sleep, Functional disability, Functional gastrointestinal disorders, Pediatrics

## Abstract

**Background:**

Sleep disturbances are increasingly recognized as a common problem for children and adolescents with chronic pain conditions, but little is known about the prevalence, type, and impact of sleep problems in pediatric functional gastrointestinal disorders (FGIDs). The objectives of the current study were two-fold: 1) to describe the pattern of sleep disturbances reported in a large sample of children and adolescents with FGIDs; and, 2) to explore the impact of sleep by examining the inter-relationships between sleep disturbance, physical symptoms, emotional problems, and functional disability in this population.

**Methods:**

Over a 3-year period, 283 children aged 8–17 years who were diagnosed with an FGID and a primary caretaker independently completed questionnaires regarding sleep, emotional functioning, physical symptoms, and functional disability during an initial evaluation for chronic abdominal pain at a pediatric tertiary care center. A verbal review of systems also was collected at that time. Descriptive statistics were used to characterize the pattern of sleep disturbances reported, while structural equation modeling (SEM) was employed to test theorized meditational relationships between sleep and functional disability through physical and emotional symptoms.

**Results:**

Clinically significant elevations in sleep problems were found in 45% of the sample, with difficulties related to sleep onset and maintenance being most common. No difference was seen by specific FGID or by sex, although adolescents were more likely to have sleep onset issues than younger children. Sleep problems were positively associated with functional disability and physical symptoms fully mediated this relationship. Emotional symptoms, while associated with sleep problems, evidenced no direct link to functional disability.

**Conclusions:**

Sleep problems are common in pediatric FGIDs and are associated with functional disability through their impact on physical symptoms. Treatments targeting sleep are likely to be beneficial in improving physical symptoms and, ultimately, daily function in pediatric FGIDs.

## Background

Chronic or recurrent abdominal pain, historically referred to as “RAP,” is the most common chronic pain entity in children and affects an estimated 10-20% of school-aged children and adolescents [1]. Only a small fraction of children with chronic abdominal pain are found to have an obvious organic cause for their pain; the vast majority of the remaining group can be diagnosed with a functional gastrointestinal disorder (FGID) based on the pattern of symptoms, with the two most common being functional dyspepsia (FD) and irritable bowel syndrome (IBS) [2,3]. Although children with FGIDs, by definition, have no obvious organic etiology sufficient to explain their symptoms, these children still experience decreases in quality of life that are comparable to children with identifiable organic diseases such as inflammatory bowel disease and gastroesophageal reflux [4]. Thus, daily functioning may be particularly important to assess as an outcome.

Over the past decade, improvements in diagnostic classification (i.e., Rome Criteria) and advancements in technology have contributed to increased investigation and understanding of the complex etiology of chronic abdominal pain. A biopsychosocial model has evolved which suggests that pain occurs as a result of varying contributions from, and interactions between, biological, psychological, and social factors [5]. Sleep is one area that exists at the intersection of biology, psychology, and environment. As a result, its role in the onset and maintenance of chronic abdominal pain broadly, and FGIDs specifically, is of great theoretical and clinical interest.

Historically, abdominal pain that interferes with normal sleep patterns or awakens the patient at night has been considered as suggestive of organic diseases, even though there is little evidence to support this concept [6]. In fact, sleep disturbances are increasingly recognized as a common problem for children and adolescents with chronic pain conditions [7]. However, research on sleep in children with chronic abdominal pain, specifically, is limited. A few studies have found that children with abdominal pain self-report higher levels of sleep disturbance than healthy controls, particularly in the areas of sleep onset/maintenance and excessive daytime sleepiness [8,9]. Consistent with this, studies have estimated the prevalence of poor sleep at 25-30% for adults with FGIDs [10-13]. No current estimate is available regarding the prevalence of sleep problems in children with FGIDs.

Sleep problems may play a major contributing role in the maintenance of chronic or recurrent pain conditions, negatively impacting daily function in a variety of ways. In the broader population of middle school children, daytime sleepiness has been associated with functional disability in the form of high rates of absenteeism, low school achievement, and low school enjoyment [14]. Beyond the impact on school, children with sleep problems have been found to have poorer parent-reported quality of life across a variety of domains than published norms for healthy peers [15]. Disrupted sleep also has been associated with higher levels of emotional problems such as anxiety and depression [16,17], and has been theorized, indirectly, to lower a child’s pain tolerance, interfere with effective use of coping skills, and increase functional disability [9,18]. Adequate sleep, in contrast, appears to directly promote tissue healing, immune function, and the body’s natural analgesic efforts, which can aid in both pain relief and recovery [19,20]. Thus, not only does sleep appear to exert an influence on daily function in children and adolescents, but this influence may occur via either a physical or emotional pathway. Clearly, these pathways from sleep to disability also may be relevant to the population of children with chronic pain, including those with FGIDs.

Consistent with this, preliminary work done with a broad array of pediatric chronic pain populations has documented linkages between sleep disturbance, physical symptoms, emotional problems, and functional disability [7,9,21]. However, to date, the inter-relationships among these variables have not been examined simultaneously within a single statistical model. In particular, the theorized mediating effects of physical and emotional symptoms in explaining the relationship between sleep disturbance and functional disability have not been examined. It will be critical to better understand the specific relationships among these variables in children with FGIDs in order to identify the most appropriate and effective targets for clinical intervention.

To this end, the current study had two primary aims: 1) to describe the pattern of sleep disturbances in children and adolescents with FGIDs; and, 2) to examine the inter-relationships between sleep disturbance, physical symptoms, emotional problems, and functional disability in children and adolescents with FGIDs. Based on the available theoretical and empirical literature, we hypothesized that sleep problems would be positively associated with functional disability and, further, that emotional and physical symptoms would mediate this relationship.

## Methods

### Participants

Participants included 283 children (M=12.0±2.5 years) and a primary caretaker (91% mothers) recruited from a single pediatric gastroenterology clinic specializing in the evaluation and treatment of children with recurrent abdominal pain. The clinic is housed within a tertiary care facility in a large Midwestern city. Consistent with the demographics of the clinic population, participants were mostly female (65%) and Caucasian (85%). All participants were referred for evaluation of abdominal pain of at least 12 weeks duration with over half of the children reporting pain for greater than a year. All participants met Rome II criteria for an FGID [22]. Diagnoses included functional dyspepsia (FD; 56%), irritable bowel syndrome (IBS; 9%), functional abdominal pain syndrome (FAPS; 2%), or both FD and IBS (33%). The gastroenterologist for the clinic (CF) made this diagnosis based on an extensive history and physical exam conducted with each child and family during the initial evaluation.

### Design and procedure

Over a 3-year period, study nurses approached 400 consecutive patients (ages 8–17 years) and their caretakers during their initial visit to the abdominal pain clinic. Patients with known organic gastrointestinal disease, previous abdominal surgery, or other significant chronic illness were excluded. Families were required to be English-speaking. If the patient was eligible and the family expressed interest, parental informed consent/permission and child assent were obtained. Approximately 75% of eligible families agreed to participate. The majority of nonparticipants cited lack of time as the reason for refusal. This study was completed as part of a long (2–3 hour) evaluation visit and nonparticipating families did not appear systematically different from consenting families. Of the original 300 participants completing the study, 17 children were excluded from analyses when organic findings later were discovered via endoscopy with biopsy, yielding a final sample of 283 participating families.

Children and caretakers completed study questionnaires independently in separate exam rooms during their initial evaluation visit, prior to receiving an FGID diagnosis. Standardized instructions were provided by study nurses. Research was carried out in compliance with the Helsinki Declaration and all procedures were approved by the Pediatric Institutional Review Board of The Children’s Mercy Hospital.

### Measures

The *Behavior Assessment System for Children (BASC)*[23] is a questionnaire assessing psychological functioning in youth, with different versions for children (ages 8–11), adolescents (ages 12–18), and parents (different versions for parents of children ages 6–11 and 12–18) to complete. The BASC scales provide standardized descriptions of problem behaviors and competencies. All versions of the BASC generate individual subscales for Anxiety and Depression. A Somatization subscale also is generated for both parent-report versions of the BASC, as well as for the adolescent self-report version. The BASC has demonstrated criterion-related and construct validity, has good internal consistency for most of the individual subscales, and is widely used in both clinical and research settings [23].

The *Functional Disability Inventory (FDI*) [24] is a 15 item questionnaire assessing a child’s perceived activity limitations across home, school, recreation, and social domains. Separate versions are available for parents and children, with both versions asking how much physical trouble or difficulty the child would have had doing a particular activity in the past few days on a 5-point scale from “No Trouble” (0) to “Impossible” (4). A total functional disability score is calculated by summing the raw scores for the individual items (range = 0–60), with higher scores reflecting greater disability. The reliability and validity of this measure in assessing functional disability has been well established [25].

The *Questionnaire on Pediatric Gastrointestinal Symptoms (QPGS*) [26] is a questionnaire assessing symptoms and disability associated with FGIDs, with separate versions available for parents and children. Included in this questionnaire are 6 items asking how often the child missed activities at school, including class work, sports, or other activities, due to specific gastrointestinal complaints (e.g., pain, vomiting, bowel issues) in the past 3 months, as well as a duplicate 6 items asking how often the child missed activities with friends or at home. Individual items are scored on a 5-point scale from “Never” (0) to “Everyday” (4) and were summed within category to provide separate total scores (range = 0–24) by reporter for missed school and missed family/friend activities.

A *review of systems (ROS)* was completed as part of the initial visit to the abdominal pain clinic. The child and the parent were asked verbally, while together in the same room, to respond “yes” or “no” to a list of symptoms that the child may have experienced in the past. Consensus on these items was obtained at the time of checklist completion. Symptoms included weight loss, weight gain, constipation, soiling, diarrhea, vomiting, heartburn, blood in stool, difficulty swallowing, nausea, liver disease, chest pain, headache, dizziness, ADHD, asthma, joint problems and allergies.

The *Sleep Disturbances Scale for Children (SDSC*) [27] is a 26-item questionnaire completed by parents to evaluate various problems related to sleep in school-aged children and adolescents. The SDSC consists of 6 factors: 1) disorders of initiating and maintaining sleep (DIMS); 2) sleep breathing disorders (SBD); 3) disorders of arousal/nightmares (DA); 4) sleep wake transition disorders (SWTD); 5) disorders of excessive somnolence (DOES); and, 6) sleep hyperhydrosis (SHY). Subscales consistent with these factors are derived by summing up the scores for individual items within each subscale. In addition, a total sleep problems score is calculated by summing the raw scores of the individual factors. The original validation study was performed on a community sample of 1000+ Italian school children with and without known sleep disorders, and norms were developed based on these samples [27]. A recent evidence-based review of subjective sleep measures classified the SDSC as “well established” in terms of psychometrics as indicated by ability to differentiate between clinical and control groups, good diagnostic accuracy, and validation of relevant subscales with actigraphy [28]. Raw scores are standardized as T-scores (*M*=50, *SD*=10). For each sleep subscale, scores >1 SD above the mean (i.e., above 60T) are considered above average and suggest the child is “at-risk” for problems in that area, while scores 2 SD above the mean (i.e., 70T) exceed the clinical cutoff and are considered abnormal.

### Statistical analysis

Descriptive statistics were calculated to characterize the sample and determine the pattern of sleep disturbance across children with FGIDs, taking into account specific demographic variables of sex, age, and ethnicity. Mean and standard deviation were used as summary statistics for continuous variables. We used chi-square, ANOVA, and t-tests to determine whether the rate of specific sleep problems varied systematically by sex, age groups (8–12 vs. 13–18), ethnicity, and FGID diagnosis with effect size estimates provided using partial Eta squared (*h*_p_^2^).

We utilized the confirmatory factor analysis (CFA) in structural equation modeling to reveal the associations among sleep problems, emotional functioning, and physical functioning and their impact on functional disability in two steps. The CFA models were constructed on theory and then evaluated by the observational data. Pearson’s correlation was determined among manifest (observed) variables. Models were constructed using four latent variables and corresponding manifest variables. Age and race/ethnicity effects were added to all manifest variables in the models and insignificant effects were removed to keep model parsimony. Models were fitted to the data and parameters were determined by maximum likelihood estimation. We report a standardized partial regression coefficient for each path to ensure that the magnitude of each factor can be directly compared with other factors in the model. Prior to the analysis, multicollinearity assessment was conducted and no independent variables in the final models had multicollinearity issues. Numerous goodness-of-fit statistics were applied to assess the validity of the proposed models.

#### STEP 1: Latent variable identification

Sleep problems, physical symptoms, emotional symptoms, and functional disability were the four latent variables considered in this study, with each latent variable indicated by two to six manifest variables.

Sleep problems were indicated by the six sleep subscales previously described: 1) disorders of initiating and maintaining sleep; 2) sleep breathing disorders; 3) disorders of arousal; 4) sleep-wake transition disorders; 5) disorders of excessive daytime somnolence; and, 6) sleep hyperhydrosis. It was moderately reliable to use the six subscale scores as indicator variables for sleep problems (Cronbach’s alpha=0.62). This was deemed acceptable given that the items included on the six indicators were considered complementary, but not overlapping, in content.

Physical symptoms were indicated by BASC Somatization subscale scores and the total number of physical symptoms endorsed on the review of systems (ROS). Given that the Somatization subscale was available on the adolescent (13–18) version of the BASC, but not on the child (8–12) version, self-report data for Somatization was not missing in a random pattern. Therefore, the self-report BASC Somatization subscale score was not included in the analysis. Only parent-report data were included as an indicator of physical symptoms. It was moderately reliable to use parent-report BASC Somatization subscale scores and the ROS total as indicator variables for physical symptoms (Cronbach’s alpha=0.67). This was deemed acceptable given that the items included on the two indicators were considered complementary, but not overlapping, in content.

Emotional symptoms were indicated by BASC Anxiety and BASC Depression subscale scores. Subscale scores based on parent- and child self-report were added together to create two more stable indicators from the original four. This increased the Cronbach’s alpha from 0.73 to 0.78 for BASC Anxiety and BASC Depression subscale scores as indicator variables for emotional symptoms.

Functional disability was indicated by summated parent- and child self-report of missed school and missed family/friend activities. An alterative scale for functional disability was considered that included the total score for the Functional Disability Inventory (FDI). However, the total score on the FDI was not strongly compatible with missed school and missed family/friend activities (Cronbach alpha=0.74); after removing the FDI as an indicator variable, the reliability between missing school and family/friend activities dramatically increased (Cronbach’s alpha=0.93). Given that the missed school and missed family/friend activities ask about the actual amount of school and other activities missed, while the FDI assesses more theoretically parent and child perceptions of how difficult an activity would have been to perform, this lack of clear concordance was not surprising. Thus, we decided to test our model first on report of actual behavior (i.e., missed school and missed family/friend activities), followed by testing model replication using more general perceptions of disability (i.e., FDI).

#### STEP 2: Priori theoretical models construction and testing

**Figure 1:**
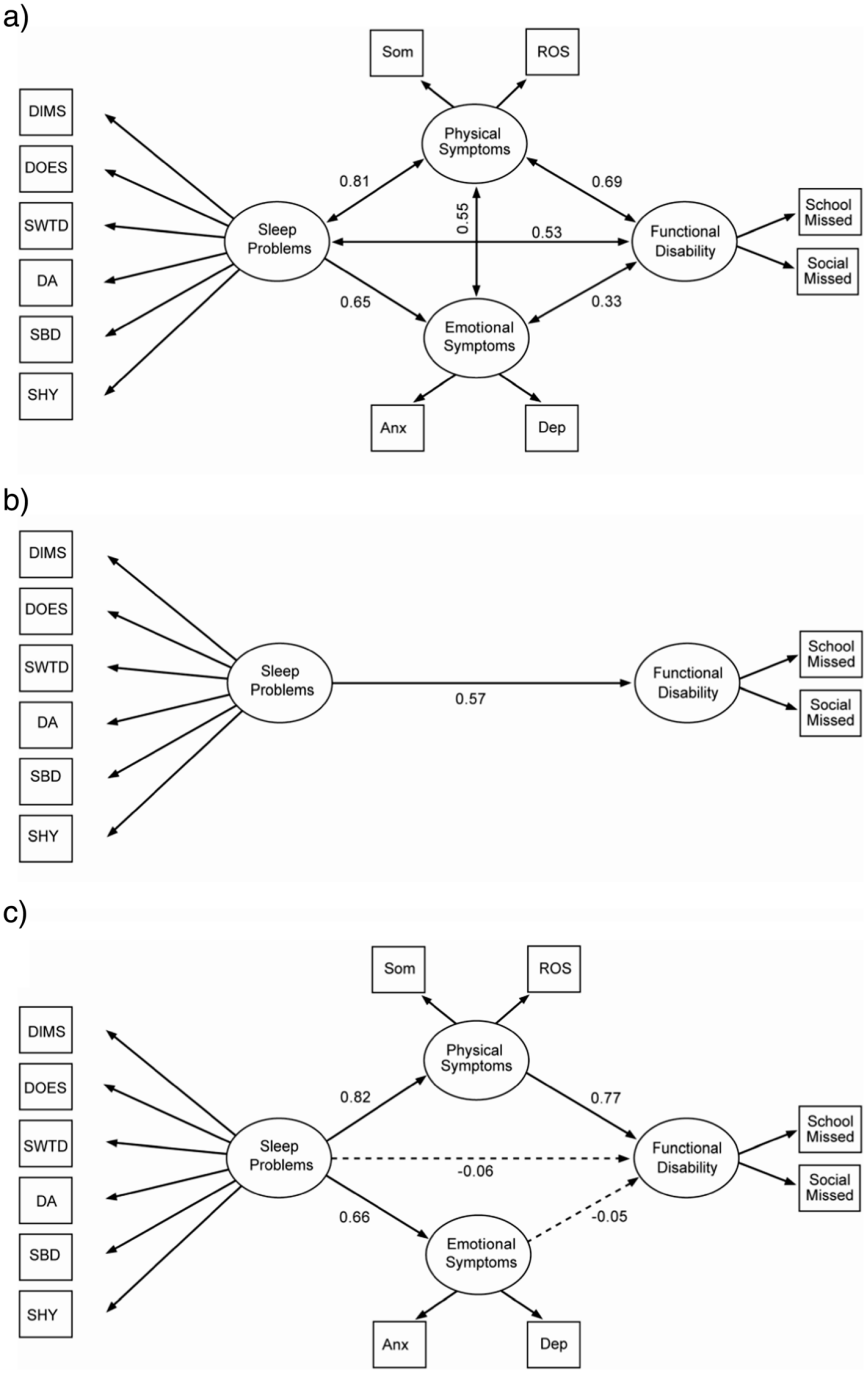
**Three major theoretical models.** Theoretical models: **a**. M1. Correlational model (*χ*^2^, p=0.18, good model fit). **b**. M2. Simple model (*χ*^2^, p=0.24, good model fit). **c**. M3. Causal model (*χ*^2^, p=0.24, good model fit). *Note.* Paths with significant partial regression coefficient or correlation coefficient (p<0.05) represented by solid lines; non-significant paths represented by dashed lines.

**Figure 2:**
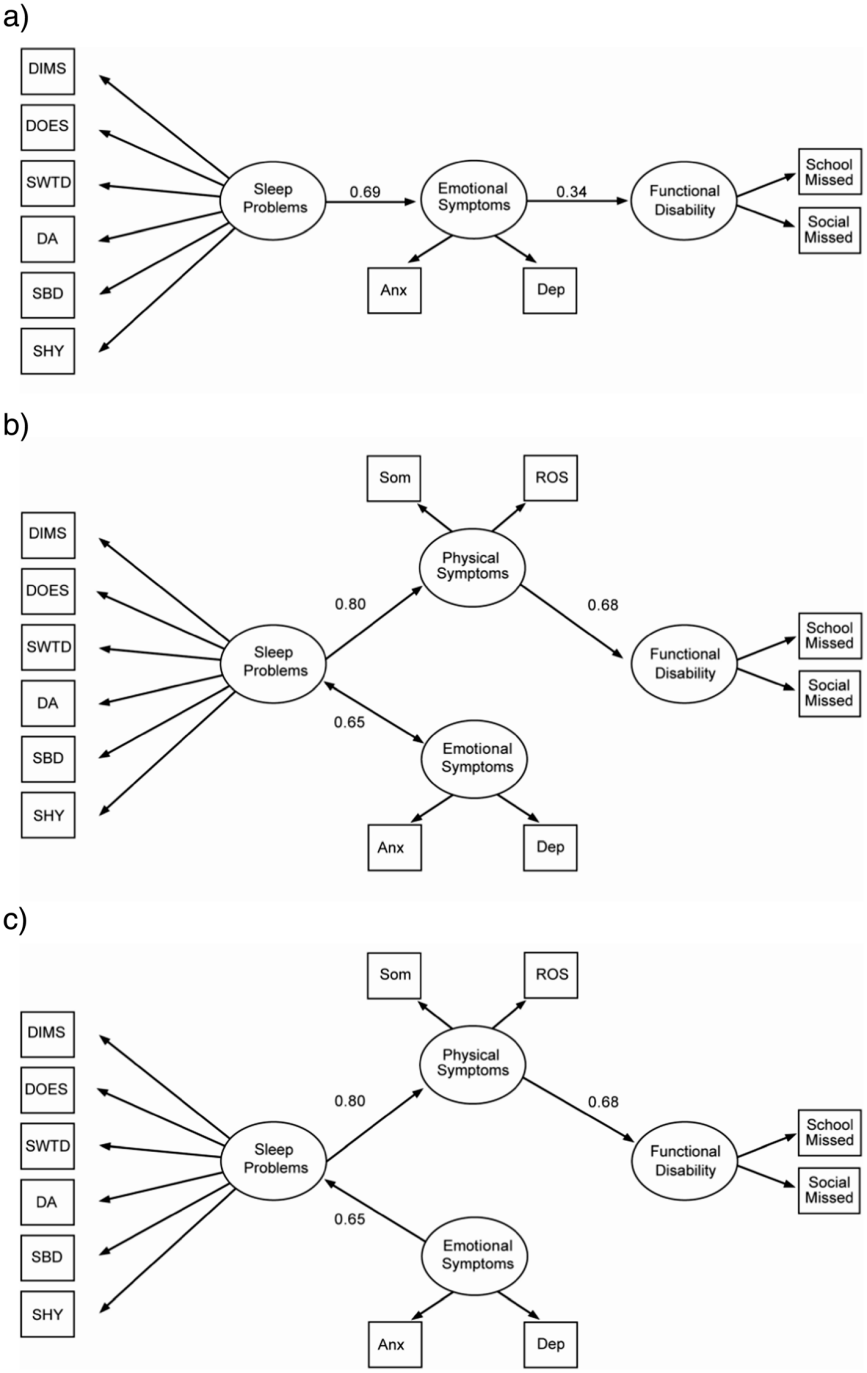
**Three extended causal models to further assess associations between sleep problems and emotional symptoms.** Extended causal models: **a**. M3.1. Pruned emotional symptoms causal model (*χ*^2^, p<0.01, poor model fit). **b**. M3.2. Emotional symptoms and sleep problems correlational model (*χ*^2^, p=0.29, good model fit). **c**. M3.3. Emotional symptoms to sleep problems causal model (*χ*^2^, p=0.29, good model fit). *Note.* Paths with significant partial regression coefficient or correlation coefficient (p<0.05) represented by solid lines; non-significant paths represented by dashed lines.

We considered three models to assess the association and impact of sleep problems to patient’s health and lifestyle: 1) a correlational model, which investigated the associations among four latent variables (see Figure 1a); 2) a simple model, which evaluated the direct path from sleep problems to functional disability (see Figure 1b); and, 3) a causal model, which considered sleep problems as the exogenous variable whose impact on functional disability is mediated by physical symptoms and emotional symptoms (see Figure 1c). Meditational variables, physical symptoms and emotional symptoms, were excluded in the simple model (see Figure 1b).

Within the present study, meditational analyses were performed in a manner consistent with the recommendations of Little, Preacher and colleagues [29]. Specifically, pathways between sleep problems and functional disability were evaluated, with and without the inclusion of the proposed mediators (emotional symptoms and physical symptoms) in the model. The strength of the associations between the predictor variables (i.e., sleep problems, emotional symptoms, and physical symptoms) and the outcome variable (i.e., functional disability) were then compared. Finally, modifications were made in accordance with both theory and empirical data, in order to identify the most parsimonious and well-fitting model. Several fit indices for the overall models are reported, including the chi-square statistics and p-values, the root mean square error of approximation (RMSEA), and the Comparative Fit Index (CFI). Good model fit was indicated by a non-significant chi-square and an RMSEA value less than 0.05, while an RMSEA value above .10 was considered indicative of a poor fit to the data [30]. Similarly, a CFI value greater than 0.9 also was considered indicative of good model fit [31]. Non-significant paths or covariance were removed from model. Model parsimony was assessed by parsimony normed fit index (PNFI), which calculated the percentages of path reduced from saturated model, and Aikaik Information Criteria (AIC). Generally, a model with a smaller AIC and a smaller (1-PCFI) is more parsimonious.

Statistical analyses were carried out by LISREL 8.8, AMOS 19, and SPSS 18 with statistical significance claimed at 95% confidence level (p<0.05). The sample size calculation was based on the 10:1 ratio against free parameters as suggested by Kline [32]. We anticipated estimating 25 parameters in parsimonious models, which equates to having a minimum of 250 participants in the study. This minimal requirement was met as we recruited 283 children and their parents for the study. Participants with incomplete data (<1%) were imputed by mean and standard deviation on the assumption that the observations were missing at random.

## Results

### Descriptive statistics and pattern of sleep disturbance

Means on the SDSC for the entire sample placed within the average range for sleep breathing disorders (51T), disorders of arousal (53T), sleep-wake transition disorders (56T), excessive daytime somnolence (57T), and sleep hyperhydrosis (48T) based on instrument norms (M=50T; SD=10T). A slight elevation was noted for difficulties with disorders of initiating and maintaining sleep (65T) and total sleep problems (60T), but both placed in “at-risk” range and did not exceed the recommended clinical cutoff (70T) for the subscale. The percent of participants exceeding the clinical cutoff for each scale is provided in Table 1. Overall, 55% of the sample had no clinically significant elevations on any sleep problem subscale. However, 26% of the sample exceeded the clinical cutoff for one sleep subscale, while 14%, 4%, and 1% exceeded the clinical cutoff for two, three, and four sleep subscales, respectively.

**Table 1:** Percent of participants exceeding the clinical cutoff for SDSC factors

	**% in Clinical range**				
**Factor**	**Total sample (n=283)**	**Girls (n=189)**	**Boys (n=94)**	**Children (n=173)**	**Teens (n=110)**
1: DIMS	29.6	30.4	28.0	23.8	38.5
2: SBD	6.5	6.5	6.5	5.3	8.3
3: DA	4.3	3.8	5.4	5.9	1.9
4: SWTD	12.6	12.5	12.9	13.0	12.0
5: DOES	15.9	13.5	20.7	13.7	19.3
6: SHY	1.4	0.5	3.2	1.8	0.9
Total Sleep Problems	22.0	22.0	22.0	19.9	25.2

No significant differences emerged based on sex, including for comparison of means on any sleep subscale (range: *h*_p_^2^ = .00-.01). However, a few differences were noted based on age. Specifically, a significantly greater percentage of teens were noted to have a clinically significant elevation for disorders of initiating and maintaining sleep (39%) than was true for younger children (24%; p<.05, FET); a higher mean score for disorders of initiating and maintaining sleep also was noted for teens (M=15.46±4.86) than for younger children (M=14.08±4.78; t=−2.32, p<.05; *h*_p_^2^ = .02). No other mean differences for age were observed on any sleep subscale (range: *h*_p_^2^ = .00-.01), nor were there any other significant differences in proportion exceeding the clinical cutoff for total sleep problems or any of the individual sleep subscales. Consistent with the clinic population from which it was drawn, the sample was ethnically homogeneous, being predominantly White (85%) with the remaining 15% split between several other categories (6% African American, 6% Hispanic, 1% Native American, 1% Asian, 1 % Other). Given problems with unequal distribution and small cell sizes, sleep problems were not compared across ethnic group, but ethnicity was considered empirically in model building.

Because of a low frequency of children with functional abdominal pain syndrome (3%), only children with functional dyspepsia (FD; 54%), irritable bowel syndrome (IBS; 11%), or both (FD+IBS; 32%) were retained for evaluation of sleep issues by specific FGID. Sleep issues did not vary systematically by FGID in the current sample for total sleep problems (*h*_p_^2^ = .01) or for any of the individual sleep subscales (range: *h*_p_^2^ = .00-.02). In addition, no significant differences were found based on FGID in proportion exceeding the clinical cutoff for total sleep problems or any of the individual sleep subscales.

## SEM results

The first step in SEM analyses involved testing the three major theoretical models outlined in Figure 1. The correlational model (M1; Figure 1a), which allowed the four latent variables to correlate freely with one another, indicated a close fit with a non-significant chi-square value (p=.18), an RMSEA of .02, and a CFI of .99 (see Table 2 for complete list of fit indices by model). The results of the correlational model indicate that the four latent variables were correlated. Sleep problems and physical symptoms had the highest correlation (rho=0.82, strong correlation) while functional disability and emotional symptoms had the lowest correlation (rho=0.39, weak correlation). To further assess the associations among four latent variables, we considered models with and without mediation variables. The simple model (M2; Figure 1b), which evaluated the direct path from sleep problems to functional disability without inclusion of any mediators, similarly indicated a close fit with a non-significant chi-square (p=.24), an RMSEA of .02, and a CFI of .99. The results of the simple model indicate that there was a significant path from sleep problems to functional disability. Finally, the causal model (M3; Figure 1c), which specified directional paths linking sleep problems to functional disability through the mediators of physical and emotional symptoms, also evidenced a close fit with a non-significant chi-square (p=.24), an RMSEA of .02, and a CFI of .99. It is very interesting to note that after introducing the mediation variables, the direct path from sleep problems to functional disability was not significant in the causal model. Results from the simple and causal models jointly suggest that the impact of sleep problems on functional disability was medicated significantly by physical symptoms.

**Table 2:** Measurements of model fit for the structure models

**Model**		**Chi Square Test**			**Baseline comparison**	**Departure from saturated models**		**Parsimony**		**Conclusion**
		***χ***^***2***^	**DF**	P-value (NS*)	CFI (>0.9*)	RMSEA (90% CI) (<0.05*)	p-value (NS*)	**1-PCFI**	**AIC**	
**Three Major Theoretical Models**	M1. Correlational model (Figure 1a)	76.4	66	0.18 NS	0.99	0.02 (0–0.04).	0.99 NS	0.38	182	Good fit
	M2. Simple model (Figure 1b)	36.2	31	0.24 NS	0.99	0.02 (0–0.04)	0.93 NS	0.46	104	Good Fit
	M3. Causal model (Figure 1c)	77.0	69	0.24 NS	0.99	0.02 (0–0.04)	0.99 NS	0.35	177	Good Fit
**Three Extended Causal Models** (Revision of M3 to further investigate association between emotional symptoms and sleep problems)	M3.1. Pruned emotional symptoms causal model (Figure 2a)	75.5	48	<0.01	0.96	0.05 (0.02-0.06)	0.64	0.41	160	Poor Fit**
	M3.2. Emotional symptoms and sleep problems correlational model (Figure 2b)	75.1	69	0.29 NS	0.99	0.02 (0–0.04)	0.99 NS	0.35	175	Good Fit
	M3.3. Emotional symptoms to sleep problems causal model (Figure 2c)	75.1	69	0.29 NS	0.99	0.02 (0–0.04)	0.99 NS	0.35	175	Good fit
**Five Replicated Models** (Using an alternative scale for functional disability)	M1 Replicate	75.1	58	0.07	0.97	0.03 (0–0.05)	0.93 NS	0.38	167	Good fit
	M2 Replicate	46	33	0.06	0.94	0.04 (0–0.06)	0.78 NS	0.44	110	Good fit
	M3 Replicate	89	71	0.07	0.97	0.03 (0–0.05)	0.97 NS	0.34	185	Good fit
	M3.2 Replicate	89	71	0.07	0.97	0.03 (0–0.05)	0.97 NS	0.34	185	Good fit
	M 3.3 Replicate	89	71	0.07	0.97	0.03 (0–0.05)	0.97 NS	0.34	185	Good fit

*Note.* **A poor fit of M3.1 counter proves the validity of the causal model M3.

As a second step, the relationship between emotional symptoms and functional disability was examined further via three extended causal models labeled as M3.1, M3.2, and M3.3 (Figure 2; see Table 2 for complete list of fit indices by model). First, to rule out the possibility of multicollinearity between physical symptoms and emotional symptoms causing the observed disconnect between emotional symptoms and functional disability, the mediational relationship linking sleep problems to functional disability through emotional symptoms was retested by removing physical symptoms in M3.1 (Figure 2a). A poor fit with a significant chi-square test (p<0.01) indicates that the hypothesized path from sleep problems to functional disability mediated by emotional symptoms was not substantiated by our data, which confirmed the original causal findings (M3; Figure 1c). An alternative model then was considered, in which the directional path from sleep problems to emotional symptoms was removed; instead, sleep problems and emotional symptoms were allowed to correlate in M3.2 (Figure 2b). A close fit was observed with a non-significant chi-square (p=.29), an RMSEA of .02, and a CFI of .99. Finally, the directional path from emotional symptoms to sleep was reversed, placing both sleep problems and physical symptoms as sequential mediators of the relationship between emotional symptoms and functional disability in M3.3 (Figure 2c). A close fit was achieved with a non-significant chi-square (p=.29), an RMSEA of .02, and a CFI of .99. The results of M3.2 and M3.3 suggest that, although emotional symptoms might correlate with or have an impact on sleep problems, the impact of sleep problems on functional disability is not mediated by emotional symptoms.

In the third step, the three major theoretical models (M1, M2 and M3) and two extended causal models (M3.2 and M3.3) that evidenced close fit were replicated using an alternative scale for functional disability that involved the Functional Disability Inventory (see Table 2 for complete list of fit indices by model). All five models demonstrated close fit to the data, with non-significant chi-squares, RMSEA values of <.05, and CFI values >.90, which confirmed our original analysis.

## Discussion

The current study aimed to describe the pattern of sleep disturbances and examine the relationship between sleep disturbance, physical symptoms, emotional problems, and functional disability in children and adolescents with FGIDs. Consistent with rates of sleep disturbance reported in previous studies of children with chronic pain more generally [33], approximately half (45%) of children and teens with FGIDs in this study were noted to have a clinical elevation on at least one sleep subscale. While the lack of a health comparison group is a limitation of the current work, a review by Owens [34] concluded that only about a quarter (25%) of children from the general population experience some type of sleep problem during their childhood [34]. Further, approximately 20% of the children and teens with FGIDs in the current study were identified as experiencing problems across more than one sleep domain. Problems related to sleep onset and maintenance were most commonly reported, and teens were noted to have a higher rate of difficulty in this area as compared to children. However, sleep problems were unrelated to any specific FGID diagnosis in our study, appearing to be a more universal phenomenon for children with chronic abdominal pain.

In addition to sleep problems being common in this population, our study found that sleep problems were positively associated with functional disability, as expected. However, mixed results were found with regard to the role of physical and emotional symptoms as proposed mediators of this relationship. Sleep problems predicted both emotional and physical symptoms in our causal model, but only physical symptoms had a direct impact on functional disability. In fact, physical symptoms fully mediated the relationship between sleep problems and problems in daily function. This finding, initially based on the estimated frequency of missed school and other activities, was replicated using the more subjective perception of functional disability afforded by the FDI.

In contrast, no direct link was found between emotional symptoms and functional disability in the current study. Examination of various extended models served to confirm that emotional symptoms do not function as a mediator of the relationship between sleep problems and functional disability, even if physical symptoms are removed from the model. It is possible that emotional symptoms are a secondary clinical outcome in themselves; in other words, both emotional problems and functional disability may result from a common source (i.e., sleep problems), but have no direct relationship or impact on one another. However, given that correlations exist among all four of the latent factors specified in model building, it remains possible that emotional symptoms do impact functional disability, but that this occurs in a more complex and indirect fashion than the causal model originally proposed. For example, our data are consistent with the possibility that sleep problems and emotional symptoms may engage in a bidirectional interaction or, alternatively, that emotional symptoms may cause sleep disruption unilaterally. In either case, emotional symptoms would contribute to functional disability via a circuitous route through physical symptoms.

A few limitations to this study are worth noting. The cross-sectional nature of this data is a relative weakness. While SEM is able to test whether directional, or causal, relationships are possible based on a particular set of data, a definitive test of causality would require a large, longitudinal dataset in which, for instance, the impact of sleep problems at one time point could be assessed on physical functioning, emotional functioning, and/or functional disability at later time points. Future longitudinal work also may allow for examining the impact of interventions to target particular components of the model on functional disability. In this manner, the most appropriate and powerful target(s) for intervention efforts may be identified.

The use of a brief and targeted questionnaire for assessing sleep problems is both a strength and weakness of the current work. This type of assessment has been recommended to enhance screening efforts of primary care and other specialty area practitioners, as it is inexpensive, easily administered, and shows reasonable sensitivity and specificity in identifying sleep disruptions [35]. In addition, the specific measure used in this study (the SDSC) has been validated with actigraphy for relevant subscales. As such, the clinical utility is high. However, some studies have found that different relationships between sleep problems and functional disability based on whether subjective or objective methods are used to determine sleep disturbance. Specifically, self-report of sleep problems has been associated with functional limitations in adults with FGIDs, while no such association is seen when objective sleep measures are employed. In fact, some studies with adults with FGIDs call into question whether adults with FGIDs actually experience sleep problems at a higher rate than healthy controls. Parent report, likely based on a combination of child self-report and more objective observation of sleep habits, may arguably be less subjective than the more pure self-report provided by adults. However, replicating the current study findings with an objective measure of sleep quality, efficiency, and/or duration would certainly be useful in further substantiating findings.

The large sample, inclusion of multi-informant data, and simultaneous consideration of several latent variables are strengths of the current work. Using both parent and child perspectives in defining the latent variables increases the likelihood of measuring the intended construct, while placing these constructs within a single model helped better define the complex relationships that contribute to functional disability in children with FGIDs. In addition, the inclusion of two different ways of measuring functional disability allowed for replication of model testing that enhances confidence in the relationships identified.

## Conclusions

Clinically, results from this study seem to suggest that intervening with emotional symptoms may not be the most direct pathway to improving daily function in children with FGIDs. Instead, both sleep problems and physical symptoms could be appropriate, and potentially more direct, treatment targets for efforts to reduce functional disability in this population. Given the biopsychosocial model underlying current understanding of pediatric FGIDs, as well as the complex correlations noted among the variables in this study, it may be that simultaneously targeting several areas of difficulty as part of a comprehensive treatment plan may be more effective than focusing effort on a single area in improving daily function.

## Abbreviations

FGID: Functional gastrointestinal disorder; SEM: Structural equation modeling; FD: Functional dyspepsia; IBS: Irritable bowel syndrome; FAPS: Functional abdominal pain syndrome; BASC: Behavior Assessment System for Children; FDI: Functional Disability Inventory; QPGS: Questionnaire on Pediatric Gastrointestinal Symptoms; ROS: Review of systems; SDSC: Sleep Disturbances Scale for Children; DIMS: Disorders of initiating and maintaining sleep; SDB: Sleep breathing disorders; DA: Disorders of arousal/nightmares; SWTD: Sleep wake transition disorders; DOES: Disorders of excessive somnolence; SHY: Sleep hyperhydrosis; CFA: Confirmatory factor analysis; RMSEA: Root mean square error of approximation; CFI: Comparative fit index.

## Competing interests

The authors declare no conflict of interest and approve the final version of the manuscript.

## Authors’ contributions

JS contributed in terms of the original idea, protocol writing, supervising the project, data analysis, and drafting of the manuscript. CF contributed to study design and manuscript writing. HD participated in data analysis and manuscript writing. CD, PH, and JC contributed to study design and provided critical revision of the manuscript. All authors have read and approved the final version of the manuscript.

## Authors’ information

Caroline Elder Danda is now in private practice at Caroline Danda, PhD, LLC, Overland Park, Kansas; Paul E. Hyman is now at The Children’s Hospital of New Orleans, New Orleans, Louisiana; Jose T. Cocjin is now at The Children’s Mercy Hospital, Kansas City, Missouri.

## Pre-publication history

The pre-publication history for this paper can be accessed here:

http://www.biomedcentral.com/1471-230X/12/142/prepub
